# Proceedings of Terrell Medical Society

**Published:** 1896-01

**Authors:** James Orr

**Affiliations:** Secretary


### Proceedings of Terrell Medical Society.

The regular meeting of Terrell Medical Society being held the first Monday in each month the last one occurred December 2d, and was largely attended, the President, Dr. W. H. Monday, being in the chair, and Dr. James Orr, Secretary.

Routine business consisted in making arrangements for the annual banquet and reunion, which takes place January 6, 1896. and Drs. James Orr, F. S. White and C. O. Mathew were appointed as the committee of arrangements.

The regular subject for discussion, "The treatment of the acute lung diseases of children," was opened by Dr. Orr in a paper outlining the general principles governing him in the treatment of these diseases. *

In discussing the paper, Dr. A. J. Stovall said he did not think alcoholic stimulants of benefit, otherwise he endorsed the paper fully.

Dr. W. P. Dumas said in treating the diseases named he usually employed counter-irritants to the chest, gave quinine freely, gives alterative doses of calomel throughout the disease, and purges freely to remove sputa which the child coughs up and swallows; prefers strychnia and digitalis as stimulants.

Other members of the society fully endorsed the paper. Dr. J. A. Anthony, however, pointing out the difficulty often met in treating these patients in open, crowded or ill-ventilated houses.

In concluding the discussion, Dr. Orr thanked the society for the cordial reception given his paper, calling attention to the fact that these were ideal cases to be approached as nearly as possible in treatment. In some open houses he found it necessary to arrange a canopy over the bed, to more properly regulate temperature or even protect the patient from noise and sudden changes.

A number of interesting cases were reported and discussed,

---

*Published herewith.—Ed.

among which was one by Dr. Orr. A primipara who had albuminuria and general anasarca prior to confinement. During labor there was a deep sulcus across the abdomen, apparently dividing the uterus into two distinct parts. There was no apparent contraction of the lower third of the uterus and notwithstanding the os was soft and fully dilated, thirty hours labor failed to bring the head through the superior straight. Pains ceasing, chloroform was administered, and a healthy child, weighing ten pounds, safely delivered. Convulsions followed recovery from the anesthetic but were promptly arrested by a hypodermic containing morphia ½ grain, Norwood's tincture 15 drops. The patient made a good recovery.

Another, by Dr. W. H. Garrett, of Forney, in which all the symptoms of typhoid fever, running a typical course, with apparent convalescence, was followed by a large abscess in the region of the kidney, which discharged through the lower bowel, and had set up a hectci condition in the patient. The question, was the abscess previously the cause of the fever, or a scondary affair,—was the feature discussed.

Other cases were, one of obscure nervous disease, by Dr. Anthony; of pneumonia with miscarriage, by Dr. Neely, of St. Elmo; two cases of malignant diphtheria in the same family, ending in death a few hours apart. Dr. White, case of peculiar fatal gunshot wound. Dr. Monday, one of sudden death in a woman in labor at full time, evidently from heart clot. In this case the family refused the Cæsarian section, notwithstanding the child was undoubtedly alive.

Gonorrhœa was selected as the subject for discussion at the next meeting, and Dr. F. S. White appointed to lead, after which adjournment took place.

JAMES ORR, M. D., Secretary.

